# Caregiver-reported good practices and their associations with care-recipient condition in dementia care: a text-mining study

**DOI:** 10.1186/s12877-026-07062-0

**Published:** 2026-01-29

**Authors:** Hiroyuki Tanaka, Masahiro Tenjin, Haruka Atosako, Yuma Nagata, Daiki Ishimaru

**Affiliations:** 1 Osaka Metropolitan University Graduate School of Rehabilitation Science, 2-1-132, Morinomiya, Joto-Ku, Osaka City, Osaka, 536-8525 Japan; 2 Graduate Student, Osaka Metropolitan University Graduate School of Rehabilitation Science, 2-1-132, Morinomiya, Joto-Ku, Osaka City, Osaka, 536-8525 Japan

**Keywords:** Dementia care, Text mining, Good practice, Person‐centered care, Nonpharmacological therapy

## Abstract

**Background:**

Dementia care requires specific “good practice” actions, which often involve tacit knowledge possessed by experienced caregivers. This study extracts and verifies caregiver-reported good practice actions that improve the condition of people with dementia and compiles them into a structured list.

**Methods:**

An online survey was conducted on Japanese care and medical professionals (nurses, care workers, therapists, and care managers). Participants answered questions on basic attributes, dementia attitude scales, dementia knowledge scales, and the Approaches to Dementia Questionnaire (ADQ) and provided free-text descriptions of good practice in six care situations: eating, mobility/transfer, hygiene, toileting, bathing/dressing, and communication Free-response comments were analyzed using KH Coder, as follows: (1) cooccurrence network analysis was performed to identify clusters of related terms, and (2) participants were divided into two groups based on the median ADQ score and distinctive terms were extracted from the high ADQ score group. Subsequently, a list of items serving as good practice was compiled, and the item list was validated using the Delphi method. A panel of 16 experts evaluated the appropriateness of each good practice item using a content validity ratio (CVR).

**Results:**

A total of 724 valid responses were analyzed (from 897 respondents; 173 were excluded). Frequent term analysis using KH Coder identified keywords such as “voice,” “explanation,” “eye contact,” “pace,” and “confirmation.” Further, co-occurring clusters included “explanation” and “menu” with respect to eating and “confirmation” and “temperature” for bathing/dressing. Correspondence analysis highlighted terms such as “understanding,” “preference,” “safety,” and “consent,” enabling the derivation of good practice items such as “explain the content of meals,” “match the pace,” “make eye contact,” and “check the water temperature.” Among 79 items, 72 satisfied the CVR threshold.

**Conclusions:**

By combining text mining and expert consensus, we created 72 validated good practice items representing tacit caregiver knowledge. These items, which are believed to reflect the principles of person-centered care, are expected to be useful in training programs on nonpharmacological interventions for caregivers of people with dementia.

## Background

Supporting the autonomy, dignity, and personhood of individuals living with dementia using “good practice” actions is essential and extends beyond symptom management [1]. The identification of good practice actions to care for and interact with people with dementia enhances frontline caregivers’ skills, preserves care recipients’ dignity, and improves the recipients’ quality of life (QoL) [2, 3]. In particular, providing support for essential basic activities of daily living (ADLs), such as eating and toileting, upholds care recipients’ dignity and self-efficacy, enhances their QoL, and reduces their confusion and anxiety [4]. These good practice actions must be clearly defined to ensure that care staff have the required practical guidance to maintain and improve care quality.

Moreover, research on Dementia Care Mapping underscores the importance of caregivers knowing how to interact with patients in everyday situations [5]. In their study, Gkioka et al. trained hospital staff in specific communication strategies for person‐centered care (PCC) and reported the subsequent occurrence of behavioral changes, highlighting the importance of structuring care actions and presenting them in a shareable format [6]. However, applying this theory in everyday practice remains challenging [7]. Although nonpharmacological interventions are required in medical and nursing care professions [8], they pose a serious issue. Despite accumulating significant practical knowledge through experience, experienced professionals cannot effectively pass the knowledge on to newer and less experienced professionals due to heavy workloads that limit mentoring time and high turnover rates that hinder tacit knowledge transfer [7, 9].

Studies have attempted to structure empirical knowledge from qualitative data using various theoretical frameworks, including PCC [7, 10]. However, these studies are based on data extracted from small samples and are highly unique; they did not provide lists of universal good practice actions, as well. Further, educational interventions have been shown to cause positive changes in staff attitudes and improve care-practice quality [11, 12]. Therefore, there is a growing need to formalize successful care experiences into specific, observable practices that can be systematically identified and evaluated. Previous studies have shown that dementia related knowledge and attitudes among caregivers are important factors influencing the quality of care. For example, a large hospital-based survey found that staff with higher dementia knowledge and more positive attitudes were more prepared to implement person-centered dementia care [13].

Accordingly, the present study aimed to identify caregiver-reported good practices in dementia care. The specific objectives were: (1) to extract and organize good practices from free-text responses provided by healthcare professionals, and (2) to validate these practices through expert consensus.

## Methods

This was a cross-sectional, text-mining-based study conducted among Japanese healthcare professionals recruited through an online research panel.

### Participants

The study’s participants were Japanese healthcare professionals, particularly registered nurses, care workers, physical therapists, occupational therapists, speech‐language therapists, and care managers. All the participants were recruited by the medical monitor panel maintained by Macromill, Inc. (Tokyo, Japan). Eligible participants had prior experience providing health care or caregiving services to individuals with dementia. The following demographic characteristics were obtained: age, gender, “frequency of contact with people with dementia over the past month (almost every day, several times a week, once a week, less than once a week, none),” “Knowledge and understanding of dementia care (Know, Somewhat know, Neither know nor don't know, Don't know much, Don't know),” “Gathering information and studying about dementia care over the past month (doing, somewhat doing, neither doing nor not doing, not doing much, not doing).”

### Scale to measure the awareness of person-centered dementia care

#### The Approaches to dementia questionnaire

Attitudes toward dementia care were measured using the Japanese version of Approaches to Dementia Questionnaire (ADQ) [14]. The ADQ includes 19 statements and uses a 5-point Likert scale (“1 = strongly disagree” to “5 = strongly agree”) to capture respondents’ attitudes toward people with dementia. A total score (range = 19–95) and two sub-scores, “hope” (range = 8–40) and “person-centeredness” (range = 11–55), are derived. Higher ADQ scores indicate more positive attitudes toward people with dementia. Finally, the Japanese version of ADQ has acceptable reliability and validity [15]. The ADQ demonstrated high internal consistency (Cronbach’s alpha [α] = 0.84), strong test–retest reliability (r = 0.89), and construct validity supported by factor analysis identifying two dimensions—Hope and Person-Centeredness—and convergent validity through significant correlations with dementia knowledge and care style measures.

#### Description and definition of good practice

Good practice is operationally defined as the specific actions recognized and enacted by caregivers that were found successful in improving the condition of people with dementia or that enabled them to perform actions appropriate to their goals. In this study, good practice items were collected from participants in a free-response format. In particular, questions were formulated for each of the six areas of daily living, that is, eating, elimination, getting up and moving around, personal hygiene, changing clothes and bathing, and communication, and participants were asked to describe specific practical actions constituting good practice. Furthermore, the definition of “state image” used to prompt responses was created based on the Well/Ill Being (WIB) value assessment stages of dementia care mapping [5] and provided as an example in the questionnaire. Therefore, the definition of good practice includes the following elements:

- Direct care interventions or environmental adaptations that elicited smiles or positive actions from care recipients.
- Direct care interventions or environmental adaptations that alleviated undesirable states—dissatisfaction or restlessness—in care recipients.
- Direct care interventions or environmental adaptations that drew out the care recipient’s abilities, thereby facilitating smoother care.
- Direct care interventions or environmental adaptations that drew out the care recipient’s abilities and enabled their use in daily living, rehabilitation, and recreational activities.

### Analysis

#### Exclusion criteria

The study’s exclusion criteria were as follows: participants’ responses on any one of the knowledge, attitude, or ADQ scales had 90% or more identical answers; data were missing in demographic items or on any of the scales; the participants failed to respond seriously or left items blank; they provided non-substantive comments or verbatim reproductions of the example text in free-response fields; they submitted duplicate surveys; or their responses contained obvious typographical errors or omissions.

#### Descriptive statistics

Descriptive statistics were calculated for basic attributes; each questionnaire item, and the dementia attitude scale, knowledge scale, and ADQ total score. For the evaluation scales, the mean and standard deviation (± SD) values were calculated.

#### Qualitative analysis of free descriptions

Free description data were analyzed using KH Coder (Version 3) (https://khcoder.net/en/), a software that quantitatively analyzes text data and enables users to obtain the overall picture of the data, extract characteristic words, and visualize the relationships among words [16]. This software is widely used in international papers [17], and the authors have experience using it for data analysis [18].

Quantitative content analysis is a method in which open-ended data are quantified into numeric values by identifying specific keywords and patterns, and then analyzed using statistical techniques. The purpose is to clarify trends and relationships in the data using objective numerical evidence [16, 17]. We used this approach as an objective and reproducible method to avoid the effect of researchers’ subjective perceptions on results.

#### Identification of frequent keywords for good practice

The collected free-text data were converted to the Microsoft Excel format and analyzed using KH Coder to extract vocabulary, such as nouns, verbs, and adjectives, through morphological analysis. The top 50 most frequently occurring words were calculated for each of the six care scenarios and indicated as important keywords for good practice. However, words such as “eating,” which completely matched the situation, were removed from the list of extracted words to avoid hindering the analysis.

### Structural characteristics of good practice items

Cooccurrence network and correspondence analyses were performed to list items. These methods involved a multivariate analysis on frequently used words in qualitative data and produced a cooccurrence network to ensure the connections among words in a cluster, which enabled us to search for the concepts and clusters included in the data [17, 18]. The Jaccard coefficient was used as an indicator of the association between words. This coefficient assumes a value between 0 and 1, and any value exceeding 0.2 indicates a strong association.

Correspondence analysis is a technique that visualizes relationships between extracted terms and other variables in a scatterplot. ADQ measures awareness and understanding of person-centered dementia care. Since dividing participants into high- and low-ADQ groups enables the extraction of words more strongly associated with effective good practices, in this study, participants were divided into high- and low-ADQ groups by a median split of their total ADQ scores, and correspondence analysis was conducted on the 20 most frequent terms to identify each group’s characteristics. In the resulting scatterplot, terms positioned close to the origin indicate the words common to both groups, whereas the terms located farther represent the words that were characteristic of one group or the other.

We used these analytical methods to visualize semantic connections based on the cooccurrence of words and identified the structural characteristics of good practice for each situation.

In the cooccurrence network diagram and correspondence analysis diagram, larger circles in the figures indicate higher frequency of occurrence.

### Development of the good practice item list

We created a list of items by confirming the collected raw data to ensure that at least one item in each category had a Jaccard coefficient of 0.2 or higher in the cooccurrence network, constituting a good practice at the action level. Further, in the correspondence analysis, we extracted characteristic words from the high- and low-score groups; our focus was on the words that were characteristic of the high-ADQ score group. While confirming the raw data, we created a list of items for each word, referring to the words in the high-score group, to enable their use as good practice at the action level.

In other words, we reviewed the words with high scores in the correspondence analysis for each cooccurrence network category. Subsequently, we checked all the raw data of the extracted words written by participants and, based on the vocabulary and phrases that they considered practically useful, we created a list of good practice items for each situation. This process enabled us to expand the simple frequency analysis of words and analysis of structural characteristics to identify the specific actions underlying participants’ descriptions of “successful care experiences” and “tacit knowledge of dementia care.”

### Validity verification of good practice items

The Delphi method was used to examine the validity of the good practice items extracted from the aforementioned free descriptions. The subjects were nurses and occupational therapists with master's or doctoral degrees who were involved in dementia care and were conducting research on dementia. Occupational therapists in Japan are recognized as specialists in both dementia care and activities of daily living (ADL); they were intentionally prioritized to evaluate the practical applicability of the good practice items. Delphi panelists were not drawn from the original participants. They were recruited separately through a snowball sampling approach.

They completed a questionnaire survey. For each good practice item, respondents had to select one of the following three options: “appropriate,” “neither appropriate nor inappropriate,” or “inappropriate.” Subsequently, the “content validity ratio” (CVR) was calculated for each item using the following formula [19]:

$$\mathrm{CVR}=\left(\mathrm{Ne}-\mathrm{N}/2\right)/\left(\mathrm{N}/2\right)$$

where Ne is the number of evaluators who rated the item appropriate, and N is the total number of evaluators. The items with CVR values equal to or more than 0.75 were considered valid.

### Ethical considerations

This study was conducted with the prior informed consent of participants, who were notified that participation was voluntary and data would be destroyed upon analysis completion. Ethical approval for the study was obtained from the Ethics Review Committee of the Graduate School of Rehabilitation Sciences, Osaka Metropolitan University, Japan (approval number: 2021–210) and conducted in accordance with the Helsinki Declaration and its later amendments. Clinical trial number: not applicable.

## Results

### Participant characteristics

Among the 897 survey respondents, 173 were excluded from the analysis based on the exclusion criteria. Accordingly, the analysis considered 724 valid cases. The sample comprised 225 men and 499 women, aged 20–68 years. The valid cases included 179 nurses, 188 care workers, 129 physical therapists, 135 occupational therapists, 50 speech language pathologists, and 43 care managers. The respondents’ mean professional experience was 12.8 years (SD = 8.3). Regarding facility type, 273, 65, 288, 65, and 33 participants worked in hospitals, clinics, care facilities, community-based settings, and other facilities, respectively. According to international classification standards, hospitals in this survey largely correspond to secondary and tertiary care, clinics represent primary care, and care facilities and community-based services represent long-term and community-based care, respectively. For contact frequency with people with dementia, 629 (86.9%) reported interacting almost daily or several times a week. Finally, regarding self-perceived dementia care knowledge, 524 (72.4%) reported that they “know” or “somewhat know” about dementia care. Tables 1 and 2 presents more participant characteristics.

**Table 1:** Sociodemographic characteristics of participants

	Value
Age (years), mean ± SD	41.3 ± 10.4
Sex, n (%)	Male 225 (31.1), Female 499 (68.9)
Work experience (years), mean ± SD	12.8 ± 8.3

**Table 2:** Professional characteristics of participants

		N	%
Occupation	Physical therapists	129	17.8
	Occupational therapists	135	18.6
	Speech therapists	50	6.9
	Care managers	43	5.9
	Nurses	179	24.7
	Care workers	188	26.0
Workplace facility	Hospitals (secondary/tertiary care)	273	37.7
	Workplace facility: Clinics (primary care)	65	9.0
	Nursing care facilities	288	39.8
	Community-based offices	65	9.0
	Other	33	4.6
Frequency of contact for the past month	Almost every day	487	67.3
	Several times a week	142	19.6
	Once a week	14	2.2
	Less than once a week	31	5.0
	No contact	50	8.0
Knowledge of dementia care	Know	142	19.7
	Somewhat know	382	47.2
	Neither know nor don’t know	134	21.5
	Don’t know much	51	8.1
	Don’t know	15	2.4
Gathering information about dementia over the past month	Doing	123	19.7
	Somewhat doing	295	47.2
	Neither doing nor not doing	173	27.7
	Not doing much	94	15.1
	: Not doing	39	6.3
ADQ (Approach to Dementia Questionnaire), range 19–95, mean ± SD		68.3	7.9

Values are presented as n (%) unless otherwise indicated

PT, physical therapist; OT, occupational therapist; ST, speech therapist; CM, care manager; Nurs., nurse; CW, care worker; ADQ, Approach to Dementia Questionnaire

### Scale measuring awareness of person-centered dementia care

The participants’ mean total score on the Japanese version of the ADQ was 68.30 ± 7.88.

### Listing good practice items

#### Frequently used words

Based on the free-text data collected from 724 healthcare and nursing professionals, the top 50 frequently used words were assessed for each of the six care scenarios by KH coders. Table 3 depicts the results.

**Table 3:** Top 50 frequently used words in each care situation

Eating		Bed mobility and transfer		Hygiene		Toileting		Bathing and dressing		Communication	
Extracted words	Number of occurrences	Extracted words	Number of occurrences	Extracted words	Number of occurrences	Extracted words	Number of occurrences	Extracted words	Number of occurrences	Extracted words	Number of occurrences
Assistance	252	Voice	284	Perform	283	Perform	209	Perform	200	Smile	248
Eating	182	Perform	241	Voice	194	Voice	205	Voice	166	Talk	140
Matching	117	Assist	184	Assistance	104	Assistance	164	Assistance	165	Voice	127
Voice	115	Action	145	Person	90	Toilet	143	Self	87	Conversation	126
Pace	71	Explain	108	Action	87	Confirm	87	Can	78	Match	98
Smile	71	Communicate	79	Self	85	Consider	72	Confirm	72	Eye contact	76
Performing	66	Individual	69	Explanation	55	Explain	61	Explain	68	Negative	70
Individual	62	Consent	65	Mirror	54	Individual	58	Wash	64	Other person	69
Eye contact	58	Adjust	61	Can	53	Shyness	58	Person	61	Conversation	67
Confirm	53	Obtain	61	Before	49	Movement	57	Action	51	Talking	65
Environment	53	Confirm	55	Care	46	Guide	49	Change clothes	48	Person	59
Negative	53	Always	48	Confirm	40	Same sex	48	Consider	39	Interact	52
Talk	52	Now	42	Washroom	38	Privacy	46	Part	39	Listen	52
Partner	50	Partner	42	Oral	34	Change	42	Communicate	34	Words	50
Conversation	50	Words	36	Self	34	Communicate	42	Pace	32	Topic	49
Communicate	49	Pace	35	Obtain	34	Go	39	Self	32	Listening attentively	39
Swallowing	45	Self	35	Communicate	33	Obtain	38	Temperature	30	Do	39
Listen	42	Before	32	Consent	28	Always	38	Mind	30	Eyes	39
Explain	40	Possible	31	Always	28	Intention	35	Situation	28	Be mindful	34
Talk	40	Understand	30	Smile	27	Consent	33	Clothes	27	Fun	33
Look	38	Pain	29	Go	26	Self	32	Match	26	Look	33
Respond	36	Move	28	Match	26	Case	30	Involve	25	Respond	30
Menu	35	Smile	27	Encourage	25	Diaper	28	Always	24	Person	26
Mouth	35	One by one	24	Involve	24	Possible	28	Assist	23	Kind	26
Concentration	33	Go	24	Now	23	Time	26	Shyness	23	Interest	23
Food	33	Instruct	22	Especially	23	Person	24	Caution	22	Atmosphere	22
Words	30	Talk	22	Pace	22	Before	24	Case	20	Family	21
Self	30	Action	21	Look	22	Mind	23	Response	20	Mind	20
Attention	30	Timing	20	Show	21	Respond	23	Same sex	20	Use	20
Sitting	29	Hand	20	Brushing teeth	20	Say	22	Privacy	19	Expression	20
Use	29	careful	20	Gentle	20	Diaper	21	Go	19	Say	18
Posture	26	Encourage	20	Situation	19	Polite	20	Before	19	Like	18
Time	26	Move	20	Watch	18	Involve	18	Obtain	19	Past	17
Hold	25	Understand	20	Talk	18	Adjust	18	Especially	18	Big	17
Encourage	25	Unnecessary	20	When	17	Curtain	17	Hot water	17	Understand	17
Patient	24	Use	20	Person	17	Urination	17	Wash	17	Awareness	16
Case	24	Talk	20	Time	16	Utilization	17	Watch over	16	Interact	16
Possible	23	Respond	19	Implement	16	Talk	17	Condition	16	Polite	16
Interact	23	Anxiety	19	Wash	16	Pants	16	Body	16	Communicate	16
Spoon	22	Guide	19	Necessary	16	Be mindful	16	Skin	16	Tone	15
Form	22	Mind	17	Part	16	Especially	16	Bath	15	Utilization	15
Mistake	22	Implement	17	Partner	15	Necessary	16	Intervene	15	Empathy	14
Person	22	Fall	17	Together	14	Refuse	15	Perform	15	Target	14
Intake	22	Ability	17	Use	14	Smile	15	Partner	15	Understand	14
Involved	21	Listen	17	Usage	14	Close	15	Encourage	15	Interact	13
Situation	21	Strength	17	Understand	14	One by one	14	Dressing/undressing	15	High	13
Tableware	21	Wheelchair	16	Words	13	Words	14	Falls	15	Create	13
Serving	21	Move	15	Life	13	Situation	14	Necessary	15	Eye contact	13
Swallowing	21	Get up	15	Understand	13	Bowel movement	14	Patient	14	Peace of mind	12
Careful	20	Always	15	Utilization	13	Gentle	14	Environment	14	Bring out	12
Front	20	Movement	15					Observation	14	Feelings	12
Eyes	20							Washable	14	Time	12
								Wear	14	Always	12
								Careful	14	Respect	12
								Listen	14	Weather	12
								Utilization	14	Being sure	12

In the eating situation, frequently used words such as “match,” “voice,” “pace,” and “smile” were extracted. Similarly, in the “mobility and transfer” situation, “voice,” “action,” “explanation,” and “consent” were extracted as frequently occurring terms, whereas, in the “personal care” situation, “voice,” “the individual,” “action,” “self,” and “explanation” were extracted. In the “elimination” situation, “voice,” “confirmation,” “consideration,” “explanation,” and “shame” were extracted as frequently occurring terms. Further, in bathing and dressing situations, frequently occurring words such as “voice,” “self,” “can,” “confirmation,” and “explanation” were extracted. Finally, in communication situations, frequently occurring words such as “smile,” “voice,” “match,” “eye contact,” and “denial” were extracted.

#### Results and the interpretation of cooccurrence networks

**Fig. 1:**
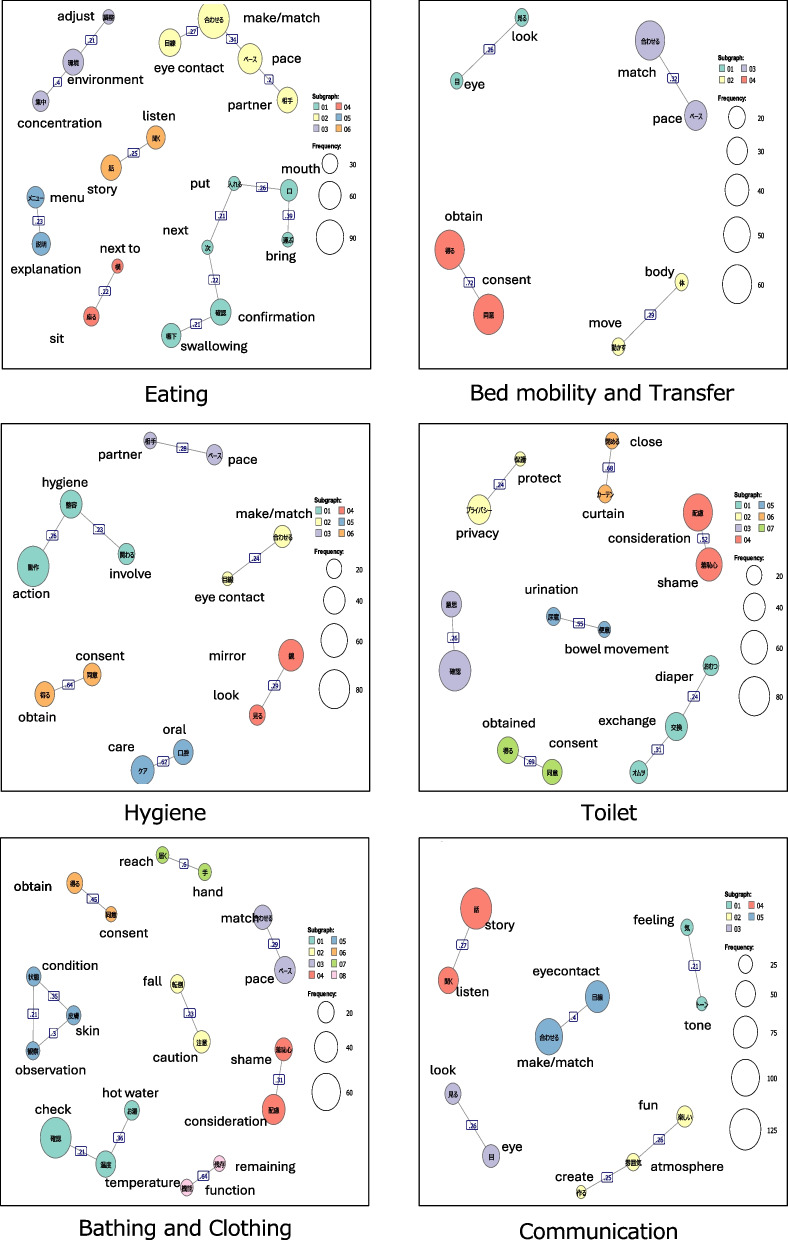
Cooccurrence network diagram for each care situation. The larger circles in the figure indicate that the words appear frequently

The visualization of the cooccurrence network structure revealed multiple common words (“smile,” “eye contact,” “wait,” “obtain consent,” etc.) in all situations. For each category with a Jaccard coefficient of 0.2 or higher in the cooccurrence network, we created a list of items that could be practiced by caregivers at the action level and selected one or two items to confirm the raw data (Fig 1).

##### Eating situation

In the eating situation, the cooccurrence network was divided into six clusters, including “explanation” and “menu.” Phrases such as “explaining the contents of the meal (menu)” were interpreted and extracted from raw data and categorized as good practice items; these are listed in Table 3.

##### Bed mobility and transfer situation

In this situation, the cooccurrence network was divided into four clusters, including “pace” and “match.” Phrases such as “match the pace” were interpreted as good practice items; they are listed in Table 3.

##### Hygiene situation

In the hygiene situation, the cooccurrence network was divided into six clusters, including “eye contact” and “made (eye contact).” Phrases such as “made eye contact” were interpreted as good practice items (Table 3).

##### Toileting situation

In the toileting situation, the cooccurrence network was divided into seven clusters, including “intention” and “confirmation.” Phrases such as “confirming the intention to use the toilet” were interpreted as good practice items (Table 3).

##### Bathing and dressing situation

In this situation, the cooccurrence network was divided into eight clusters, including “confirming,” “temperature,” and “hot water.” Phrases such as “checking the temperature of the hot water” were interpreted as good practice items (Table 3).

##### Communication situation

In the communication situation, the cooccurrence network was divided into five clusters, including “enjoyable,” “atmosphere,” and “create.” Phrases such as “creating an enjoyable atmosphere” were interpreted as good practice items (Table 3).

#### Correspondence analysis results and their interpretation

**Fig. 2:**
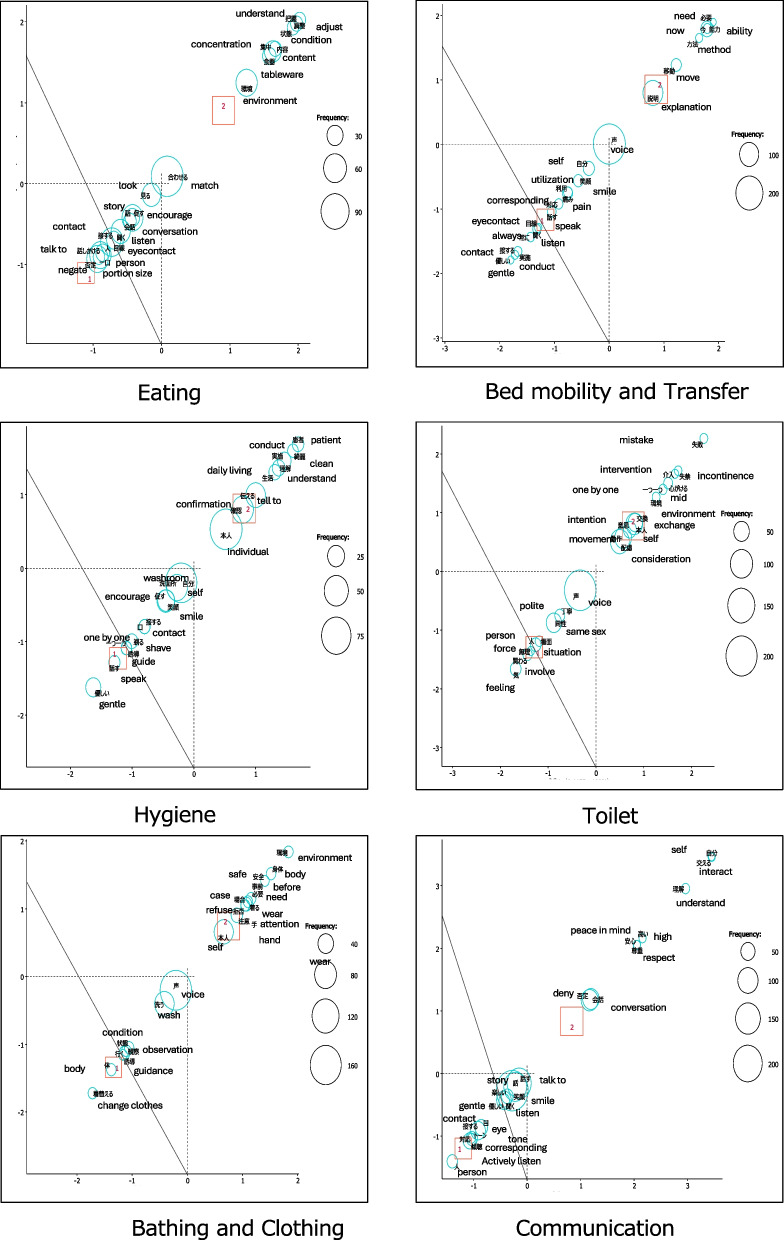
Correspondence analysis diagram for each care situation. Participants were divided into two groups based on the median Approaches to Dementia Questionnaire score (group 1: low scores; group 2: high scores), and the top 20 most frequent words were extracted. Subsequently, a correspondence analysis scatterplot was generated to identify the good practice (GP) items characteristic of the high-ADQ score group. The larger circles in the figure indicate that the words appear frequently. The X-axis (Dimension 1) and Y-axis (Dimension 2) indicate relative “distance,” with words in the positive direction being characteristic of the High-ADQ group and those in the negative direction being characteristic of the Low-ADQ group

As part of correspondence analysis, data were divided into two groups—high- and low-ADQ score groups—and characteristic words were extracted from the high‐score group. After confirming the words identified in the high-score group against the original raw data, we created a list of items that can be implemented by caregivers as good practice actions (fig 2).

##### Eating situation

For the high-ADQ score group, words such as “preference,” “understanding,” “concentration,” and “environment” were extracted. Based on these words, good practice items such as “understanding food preferences” and “creating an environment conducive to concentration” were identified (Table 3).

##### Bed mobility and transfer situation

Words such as “explanation” and “ability” were extracted. Based on these words, good practice items such as “explained each assistive action” and “utilized remaining abilities” were identified.

##### Hygiene situation

Words such as “communicate” and “understand” were extracted. Based on these words, good practice items such as “communicated that they were clean” and “communicated the purpose of hygiene and obtained consent” were identified.

##### Toileting situation

Words such as “one by one” and “intention” were extracted. Based on these words, good practice items such as “explained each assistance action” and “confirmed the intention to defecate” were identified.

##### Bathing and dressing situation

Words such as “refusal” and “safety” were extracted. Based on these words, good practice items such as “did not force the patient if they refused” and “set up a safe environment (handrails, seat arrangement, etc.)” were identified.

##### Communication situation

Words such as “understanding,” “exchanging,” and “denying” were extracted. Based on these words, good practice items such as “used gestures (body language) while talking” and “did not deny the content of the conversation.” In summary, this analysis extracted a total of 79 good practice items, with items such as “making eye contact” included in communication situations (Table 4).

**Table 4:** CVR values for each care situation

Eating			Bed mobility and transfer		Hygiene		Toileting		Bathing and clothing		Communication	
Item no	Item	CVR	Item	CVR	Item	CVR	Item	CVR	Item	CVR	Item	CVR
1	Confirmed swallowing before bringing the next item to the mouth	0.875	Matched pace (matched the other person's movements)	0.875	Made eye contact	0.625	Closed curtains and doors to ensure privacy	0.875	Checked water temperature	0.875	Created a fun-filled atmosphere	1
2	Made eye contact	0.75	Communicated the transfer’s purpose and obtained consent	1	Matched the pace (adjusted to the person's movements)	0.875	Guided the individual to the toilet	0.875	Adjusted the pace (to match the movements of the person being assisted)	0.75	Listened attentively	1
3	Matched the pace (adjusted to the person's movements)	0.875	Made eye contact	0.875	Shaving, brushing teeth, and taking care of hygiene were performed in front of the sink or using a mirror provided	0.875	Confirmed the individual's intention to use the toilet	0.875	Closed curtains and doors to ensure privacy	0.875	Made eye contact	1
4	Created an environment conducive to concentration	0.875	Listened attentively	0.875	Explained the purpose of hygiene and obtained consent	0.875	Explained the purpose of using the toilet and obtained consent	0.625	Observed the skin’s condition	0.875	Asked about topics of interest, hobbies, and life experiences	1
5	Sat down next to the person	0.625	Approached the person with a smile	1	If the person resisted, did not force them, gave them time, or took extra time	0.875	Interacted with a smile	0.75	Explained the purpose of bathing and changing clothes and obtained consent	0.875	Communicated in short, easy-to-understand words	1
6	Sat down facing the person	0	Checked for pain (facial expressions)	1	Approached the person with a smile	0.875	Encouraged cooperation where possible (utilized remaining abilities)	0.875	Explained each step of the assistance process	0.875	Interacted with a smile	1
7	Explained the meal menu	0.75	Cooperated with the other person as much as possible (utilized remaining abilities)	1	Asked for cooperation where possible (utilized remaining abilities)	0.875	Adjusted pace to match the individual's movements	0.875	Established a safe environment (handrails, seating arrangements, etc.)	0.875	Talked about family (life experiences)	0.75
8	Understood the person's food preferences	0.875	Used gestures to help the other person understand the transfer movements	1	Explained each assistive action	0.75	Conducted the procedure in an environment conducive to the individual's needs (e.g., handrail placement)	0.875	Interacted with a smile	0.875	Used gestures (body language) while talking	1
9	Did not negate the content of the conversation	0.875	Confirmed the position of the other person's arms and legs	1	Praised them for looking clean	0.875	Took care to dress and undress gently	0.875	Received assistance from someone of the same gender	0.875	Called the person by name	1
10	Interacted with a smile	0.875	Performed the transfer in a way that minimized strain	0.75	Used tools familiar to the individual in their daily life (e.g., razor, comb)	0.875	Reassured the individual not to worry about incontinence	0.25	Encouraged cooperation for the tasks the person could perform (utilizing remaining abilities)	0.875	Greeted the person	1
11	Used the same utensils the person used at home	0.75	Explained each assistance action	0.75	Used gestures to help the individual understand hygiene actions	0.875	If there was refusal, did not force the individual (did not perform the procedure against their will)	0.875	Started with individuals without paralysis (or with mild paralysis) for undressing (when dressing, started with those with paralysis) (not applicable for dementia patients)	−0.875	Did not negate the content of the conversation	1
12	Used utensils that were easy to hold and eat with	0.875					Responded with a same-sex caregiver	0.875	Avoided forcing assistance if refused (did not perform procedures against the individual's will)	0.875	Nodded; spoke loudly, slowly, and clearly	1
13	Adjusted the form of the meal	0.875					Explained each assistive action step by step	0.75			Spoke in a way that made my facial expressions clear to the other person	1
14	Adjusted the person's posture to a comfortable one for eating	0.875									Spoke face to face	0.625
15	Assisted the individual to see the food	0.875										
16	Engaged in conversation related to the meal (other than explaining the menu)	0.875										
17	Adjusted the portion size	0.875										
18	Communicated that the meal was delicious	0.75										

*CVR* Content validity ratio

### Validity verification results for good practice items using the Delphi method

Sixteen experts (15 occupational therapists and 1 nurse) participated in the validity assessment of each good practice item. Subsequently, 72 of 79 items met the CVR criterion of 0.75 or higher, and at least 13 participants evaluated them as valid. The remaining seven items did not meet the CVR criterion (Table 4).

The items that did not meet CVR criteria included specific opinions such as “The seating position during eating varies depending on the situation; so, it cannot be generalized,” which were considered practical and relevant to the clinical setting. Items that did not meet the CVR threshold were reviewed by the authors against the Delphi panelists’ stated reasons for exclusion and were removed from the final good practice list. No new items were added during this process.

## Discussion

In this study, 72 items of practical knowledge were extracted as good practice from 724 medical and care professionals involved in dementia care in six care situations. These findings have important implications in establishing a foundation for educational interventions aimed at improving dementia care quality. In existing dementia care education programs such as Humanitude, techniques like “seeing” and “touching” are emphasized as essential components of care [20]. Previous research has highlighted the importance of addressing residents by name and using positive language in communication training for nursing staff [21]. These approaches are important and overlap with several of the good practice items identified in our study. However, most existing programs focus on general communication techniques with people with dementia, whereas the present study uniquely organizes good practices by specific ADL situations, thereby providing a structured framework that can be more directly translated into everyday care practices and training modules. This situational organization may enhance applicability in clinical training because it allows staff to practice context-specific behaviors that are immediately relevant to daily caregiving tasks.

Our good practice items can be organized by ADL situation to develop training modules. These modules can form the basis of hands-on workshops and simulation exercises that allow health professionals to practice these actions and receive targeted feedback. By embedding these items into competency checklists, institutions can measure proficiency, identify gaps, and tailor future training. Future studies should assess whether such focused training reduces the behavioral and psychological symptoms of dementia, decreases staff burnout, and improves self-efficacy.

### Characteristics of participants in this study

The participants had frequent contact with people with dementia as part of their daily work and scored high on measures of PCC awareness. In this study, the mean ADQ score was 68.3 (SD = 7.9). A cross-sectional study in 15 Dutch nursing homes reported ADQ scores among healthcare professionals ranging from 65.7 to 74.2 [22]. Our participants achieved comparable scores. These participants’ characteristics support the reliability of the survey’s free-response data, as they reflect the practical knowledge of experienced, specialized professionals working in the clinical practice.

### Practical knowledge in dementia care

Dementia care settings are characterized by busy daily routines and high staff turnover, which makes it difficult for experienced staff to pass on their practical knowledge to newer staff [23]. This study indicates that structuring tacit knowledge through cooccurrence network analysis and linking it to PCC through correspondence analysis has several implications. It reveals the meaningful connections underlying high-quality practice and clarifies how the dementia care process can be translated into concrete, actionable steps. In addition, presenting these practices as a structured list makes them easier to transfer to newly recruited staff, thereby facilitating continuity of good dementia care even in settings with frequent turnover.

When listing good practice items, the items extracted in this study, smile, eye contact, and “obtaining consent,” are commonly used in any situation, and these words may be the core elements of each daily living situation, such as eating, toileting, and bathing. This may indicate that the PCC philosophy is a crucial part of the practical knowledge of healthcare and nursing staff. In particular, the fact that a list of specific actions related to the consideration for individuality and respect for autonomy, such as “understanding meal preferences” and “not forcing refusal,” was extracted from the high-ADQ score group suggests that the PCC philosophy comprises not only theoretical knowledge but also actions.

Research on PCC clarifies that the generalizability of specific care behaviors is a challenge [24]. In this study, we used text mining, a quantitative method, to extract practical knowledge, or the experiences that were considered successful by medical and nursing care professionals, and identified specific actions based on PCC principles. This approach plays an important role in linking abstract concepts to concrete practices and is consistent with earlier studies on ADL support that lead to “peace of mind” and “maintaining autonomy” [3]. It is well established that agitation often occurs during bathing or excretion [25]. The good practice list of each ADL situation can provide guidance for future educational interventions as practical nonpharmacological care for behavioral and psychological symptoms of dementia [8].

### Validity of good practice items and list refinement using the Delphi method

Expert evaluation using the Delphi method confirmed the high practicality and reliability of the extracted good practice items. The impractical items that did not meet CVR criteria were removed from the list. Therefore, this good practice list is consistent with expert opinion and has been established as a highly refined list. In addition, we clarified the basis for applying the CVR threshold. The cutoff was set at ≥ 0.75 in accordance with Lawshe’s method [19]. Notably, a recent study used a lower cutoff (≥ 0.56) with 12 experts in developing a dementia-related scale [26], whereas our study adopted the stricter criterion of ≥ 0.75 with a larger expert panel (n = 16). This comparison further supports the robustness and validity of the finalized good practice item list.

### Limitations of and future challenges posed by this study

This study has several limitations. First, since free-response data are based on respondents’ subjective opinions, they may not cover all good practice aspects. Additionally, the validity assessment using the Delphi method was conducted by a limited number of experts and did not incorporate the opinions of family caregivers and persons with dementia. Moreover, most Delphi participants were occupational therapists, which underrepresented the perspectives of other healthcare professionals, such as nurses and physiotherapists. Occupational therapists are specialists in dementia care and ADL and, therefore, highly qualified to evaluate practical aspects of good practice items. These aspects may affect the generalizability of the findings. Hence, future research must incorporate intervention studies to verify the effectiveness of good practice item lists as educational programs.

### Future developments and practical applications

Educational interventions effectively change staff attitudes and increase self-efficacy, thereby improving the quality of care practices [12]. The good practice item list obtained in this study is expected to serve as effective specific content for such educational interventions. In particular, the items can be organized by ADL situations—such as eating, toileting, and bathing—to create structured training modules. This organization can be practiced through simulation exercises. This approach is consistent with prior work that highlights respect, structured processes, and supportive environments as common themes across ADL domains [27]. Moreover, intervention studies, including communication-focused training that emphasizes brief instructions and positive language, have improved staff–resident interactions and reduced caregiver distress, supporting the applicability of our good practice items as structured training content [21].

For example, it provides practical guidance on specific content for “speaking to patients” in various situations and postures when making eye contact. Moreover, care training is reported to cause behavioral changes in healthcare professionals [28], and the good practice list can serve as specific content for similar educational interventions.

## Conclusions

This text-mining study of caregiver-reported “good practices” identified practice elements that were associated with more favorable care-recipient conditions in dementia care. These findings highlight the importance of systematically identifying effective care practices and incorporating them into staff training and individualized care planning.

## Data Availability

The survey instrument is available from the corresponding author on reasonable request. The dataset analyzed during the current study is not publicly available due to confidentiality considerations but is available from the corresponding author on reasonable request.

## Abbreviations

ADL/ADLs: Activities of Daily Living
ADQ: Approaches to Dementia Questionnaire
α: Cronbach’s Alpha (measure of internal consistency)
CVR: Content Validity Ratio
KH Coder: Quantitative Text Analysis Software developed by Koichi Higuchi
PCC: Person-Centered Care
QoL: Quality of Life
SD: Standard Deviation
WIB: Well/Ill-Being (Value Assessment stages of Dementia Care Mapping)
JSPS: Japan Society for the Promotion of Science
KAKENHI: Grants-in-Aid for Scientific Research (by JSPS)
PhD: Doctor of Philosophy
OT: Occupational Therapist
PT: Physical Therapist
ST: Speech Therapist
CM: Care Manager
Nurs: Nurse
CW: Care Worker
r: Pearson’s Correlation Coefficient (used for test–retest reliability)
n: Sample Size
N: Total Number of Evaluators (in CVR formula)
Ne: Number of Evaluators rating an item as “Appropriate” (in CVR formula)
Jaccard Coefficient: A measure of similarity between two data sets used in cooccurrence analysis

## References

[1] Serbser-Koal J, Dreyer J, Roes M. Autonomy and its relevance for the construction of personhood in dementia—a thematic synthesis. BMC Geriatr. 2024;24:255. 38486169 10.1186/s12877-024-04808-6 PMC10941450

[2] Fazio S, Pace D, Flinner J, Kallmyer B. The fundamentals of person-centered care for individuals with dementia. Gerontologist. 2018;58(Suppl 1):S10–9. 29361064 10.1093/geront/gnx122

[3] Brooker D. What is person-centered care in dementia? Rev Clin Gerontol. 2003;13:215–22.

[4] van Wijngaarden E, Wiggins RD, Jones R, et al. Good care for older people with dementia: insights from family caregivers’ narratives. J Clin Nurs. 2018;27(1–2):e145–55.

[5] Brooker D. Dementia care mapping: a review of the research literature. Gerontologist. 2005;45(Suppl 1):11–8. 16230745 10.1093/geront/45.suppl_1.11

[6] Gkioka M, Kouroupa A, Galanis P. The effectiveness of education programs for nursing staff on knowledge, attitudes and communication skills in dementia care: a systematic review and meta-analysis. J Psychiatr Ment Health Nurs. 2020;27(6):724–41.

[7] Moore L, Britten N, Lydahl Ö, Naldemirci Ö, Elam M, Wolf A. Barriers and facilitators to the implementation of person-cenetred care in different healthcare contexts. Scand J Caring Sci. 2017;31(4):662–73. 27859459 10.1111/scs.12376 PMC5724704

[8] Spector A, Orrell M, Goyder J. A systematic review of staff training interventions to reduce the behavioural and psychological symptoms of dementia. Ageing Res Rev. 2013;12(1):354–64. 22820151 10.1016/j.arr.2012.06.005

[9] Lourida I, Abbott RA, Rogers M, et al. Dissemination and implementation research in dementia care: a systematic scoping review and evidence map. BMC Geriatr. 2017;17:147. 28709402 10.1186/s12877-017-0528-y PMC5513053

[10] Kejžar A, Turunen KM. The ecosystem of human capital in care homes. Front Public Health. 2024;12:1298833. 38500729 10.3389/fpubh.2024.1298833 PMC10946669

[11] Britten N, Lydahl Ö, Naldemirci Ö, Elam M, Wolf A. Elaboration of the Gothenburg model of person-centered care. Health Expect. 2016;20(3):407–18. 27193725 10.1111/hex.12468 PMC5433540

[12] Surr CA, Gates C, Robertson S, Griffiths A, Rait G. Effective training strategies for the implementation of person-centered care in dementia: a systematic review. Int Psychogeriatr. 2017;29(2):247–62.

[13] Keogh F, Carney P, Cooke G, Manning E, McGrane N, O’Brien J, et al. Acute hospital staff’s attitudes towards dementia and perceived dementia knowledge: a cross-sectional survey in Ireland. BMC Geriatr. 2020;20:540. 32998718 10.1186/s12877-020-01783-6 PMC7526250

[14] Lintern T, Woods B. Approaches to Dementia Questionnaire. Bangor: University of Wales; 2001.

[15] Suzuki M, Mizuno Y, Greiner C, Fukahori A, Isowa T, Sakamoto R, et al. The effects of person-centered care intervention using Dementia Care Mapping in severe dementia care. Jpn J Geriatr Psychiatry. 2009;20:668–80.

[16] Higuchi K. New quantitative text analytic method and KH coder software. Jpn Sociol Rev. 2017;68:334–50.

[17] Sasaki D. Analysis of the attitude within Asia-Pacific countries towards disaster risk reduction: text mining of the official statements of 2018 Asian ministerial conference on disaster risk reduction. J Disaster Res. 2019;14(8):1024–9.

[18] Ueno K, Tanaka H, Niki K, Ueda M, Tanaka A, Yokoi K, et al. Effects of real-time VR clinical practice on reducing stigma toward dementia among students of occupational therapy: a randomized controlled trial. PCN Rep. 2023;2:e160. 38868728 10.1002/pcn5.160 PMC11114262

[19] Lawshe CH. A quantitative approach to content validity. Pers Psychol. 1975;28(4):563–75.

[20] Henriques LVL, de Melo RC, de Paiva RO. Implementation of the Humanitude Care Methodology: contribution to the quality of health care. Rev Lat Am Enferm. 2019;27:e3123. 10.1590/1518-8345.2430-3123 PMC6336364 30698221

[21] Sprangers S, Dijkstra K, Romijn-Luijten A. Communication skills training in a nursing home: effects of a brief intervention on residents and nursing aides. Clin Interv Aging. 2015;10:311–9. 25653513 10.2147/CIA.S73053 PMC4309793

[22] Gerritsen DL, van Beek APA, Woods RT, Verkaik R, de Lepeleire J, Leontjevas R, et al. Relationship of care staff attitudes with social well-being and challenging behavior of nursing home residents with dementia: a cross sectional study. Aging Ment Health. 2019;23(11):1517–23. 30409022 10.1080/13607863.2018.1506737

[23] Takeuchi Y, Kato M, Kitamura T, Toda D, Taniguchi Y, Shogenji M, et al. Development of a professional care program for nurses in dementia wards and its educational effects. Am J Alzheimers Dis Other Demen. 2020;35:1533317520950925. 32865422 10.1177/1533317520950925 PMC11005323

[24] Surr CA, Parveen S, Smith SJ, Drury M, Sass C, Burden S, et al. The barriers and facilitators to implementing dementia education and training in health and social care services: a mixed-methods study. BMC Health Serv Res. 2020;20:512. 32503536 10.1186/s12913-020-05382-4 PMC7275489

[25] Schreiner AS, Yamamoto E, Shiotani H. Agitated behavior in elderly nursing home residents with dementia in Japan. J Gerontol B Psychol Sci Soc Sci. 2000;55(3):180–6. 10.1093/geronb/55.3.p180 11833979

[26] Sharif Nia H, Pahlevan Sharif S, Khoshnavay Fomani F, Goudarzian AH, Haghdoost AA, Allen KA. Development and psychometric evaluation of the Dementia Health Literacy Scale in Iranian older adults. BMC Geriatr. 2023;23:41.36690954

[27] Prizer LP, Zimmerman S. Progressive support for activities of daily living for persons living with dementia. Gerontologist. 2018;58(Suppl_1):S74-87. 29361063 10.1093/geront/gnx103 PMC5881654

[28] Hunter KF, Dahlke S, Negrin K, Kalogirou MR, Fox M, Antonio N, et al. The feasibility of implementing education on older person care to practice on medical units: nurses’ perceptions and the influence of practice context. Int J Older People Nurs. 2019;14:e12265. 31441244 10.1111/opn.12265

